# Deep brain stimulation of the subthalamic nucleus for Parkinson’s disease: A network imaging marker of the treatment response

**DOI:** 10.21203/rs.3.rs-4178280/v1

**Published:** 2024-05-07

**Authors:** Prashin Unadkat, An Vo, Yilong Ma, Shichun Peng, Nha Nguyen, Martin Niethammer, Chris C. Tang, Vijay Dhawan, Ritesh Ramdhani, Albert Fenoy, Silvia Paola Caminiti, Daniela Perani, David Eidelberg

**Affiliations:** The Feinstein Institutes for Medical Research; The Feinstein Institutes for Medical Research; Center for Neurosciences, Institute of Molecular Medicine, The Feinstein Institutes for Medical Research, Manhasset, New York, USA.; Center for Neurosciences, Institute of Molecular Medicine, The Feinstein Institutes for Medical Research, Manhasset, New York, USA.; Albert Einstein College of Medicine; The Feinstein Institutes for Medical Research; The Feinstein Institutes for Medical Research; The Feinstein Institutes for Medical Research; Donald and Barbara Zucker School of Medicine at Hofstra/Northwell; The Feinstein Institutes for Medical Research; Vita-Salute San Raffaele University; Vita-Salute San Raffaele University; The Feinstein Institutes for Medical Research

## Abstract

Subthalamic nucleus deep brain stimulation (STN-DBS) alleviates motor symptoms of Parkinson’s disease (PD), thereby improving quality of life. However, quantitative brain markers to evaluate DBS responses and select suitable patients for surgery are lacking. Here, we used metabolic brain imaging to identify a reproducible STN-DBS network for which individual expression levels increased with stimulation in proportion to motor benefit. Of note, measurements of network expression from metabolic and BOLD imaging obtained preoperatively predicted motor outcomes determined after DBS surgery. Based on these findings, we computed network expression in 175 PD patients, with time from diagnosis ranging from 0 to 21 years, and used the resulting data to predict the outcome of a potential STN-DBS procedure. While minimal benefit was predicted for patients with early disease, the proportion of potential responders increased after 4 years. Clinically meaningful improvement with stimulation was predicted in 18.9 – 27.3% of patients depending on disease duration.

## Introduction

Parkinson’s disease (PD) is an increasingly common degenerative brain disorder characterized primarily by progressive motor dysfunction. The dopamine system is consistently affected by the disease process, and repletion of this neurotransmitter remains the mainstay of treatment in early-stage patients. However, the response to dopaminergic medication becomes less predictable over time, as patients begin to experience motor fluctuations and peak-dose dyskinesias [1]. It is at this stage where surgical management of the disease is considered. Deep brain stimulation (DBS) is a surgical procedure that involves implantation of electrodes into specific areas along abnormally functioning neural pathways through which electrical stimulation is delivered on chronic or adaptive basis [2]. Nearly three decades since the first reports of its therapeutic utility, DBS applied to the dorsolateral subthalamic nucleus (STN) motor subregions has been consistently shown to improve symptoms of akinesia, rigidity, and tremor in PD patients with moderate or advanced disease [3–7]. Moreover, targeting these areas with STN-DBS improves motor fluctuations, reduces daily levodopa dose requirements and peak-dose dyskinesias – together improving quality of life in chronically levodopa-treated PD patients [7, 8]. While DBS at other targets along motor pathways, such as the ventral intermediate (Vim) thalamic nucleus and the internal globus pallidus (GPi), has also been found to be effective for certain manifestations of PD, the STN is the most common target for this intervention [9].

The Identification of ideal candidates for STN-DBS and the appropriate timing of surgery are open issues, as is selecting the optimal position and stimulation parameters for the implanted electrodes [10]. In general, these assessments are based on dopamine responsive changes in motor scores on a standardized rating scale administered by a trained clinician [11]. However, alternative quantitative tools are needed for more objective patient selection and accurate postoperative assessment, particularly in underserved areas in which experienced clinical specialists are not readily available [12, 13].

Network analysis of metabolic brain scans obtained using [^18^F]-fluorodeoxyglucose (FDG) positron emission tomography (PET) may help provide a useful alternative for the evaluation of potential STN-DBS candidates. This general approach has been used to identify and validate disease-specific metabolic networks that are sensitive to the underlying pathological process and which may be modulated by treatment [14, 15]. These topographies, however, are not treatment-specific, and therefore not ideal for assessing targeted therapies such as STN-DBS. Whereas high expression levels for disease networks tend to be associated with worse clinical symptoms, elevated values do not necessarily denote those individuals who are more likely to respond to a given intervention.

In this study, we used a supervised form of principal component analysis termed Ordinal Trends Canonical Variates Analysis (OrT/CVA) [14–16] to identify a specific metabolic topography induced by STN stimulation in PD patients. Indeed, we identified a significant STN-DBS-related network (STN StimNet) for which stimulation-mediated changes in expression correlated with concurrent improvement in standardized motor ratings in independent patient groups. Moreover, baseline STN StimNet expression levels measured either off stimulation with FDG PET, or before DBS surgery with FDG PET or resting-state functional MRI (rs-fMRI) predicted the clinical response to STN stimulation in multiple independent cohorts. Finally, we show how STN StimNet expression can be used as a potential presurgical screening tool to identify the subset of PD patients likely to exhibit the best clinical response to this intervention.

## Results

### Network identification

**Figure 1:**
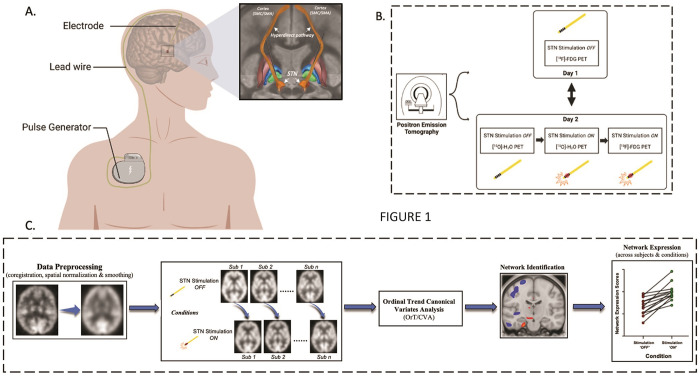
**(A)** Deep brain stimulation electrodes were stereotactically implanted into the subthalamic nucleus and tunneled subcutaneously via lead extenders to an implantable pulse generator placed on the anterior chest wall. *Inset:* 3D representation of typical bilateral deep brain stimulation (DBS) electrodes (*black*) implanted into the motor portion of the subthalamic nucleus (STN, *orange*) overlaid on adjacent coronal and axial slices from a template image using Lead-DBS [69, 77]. The volume of tissue activated (VTA, *red*), modeled from selected stimulation parameters, active contacts and the surrounding tissue shows involvement of the hyperdirect pathways (*orange tracts*) that connect the motor STN to the ipsilateral sensorimotor cortex (SMC). Adjacent basal ganglia structures anterior to the electrodes (putamen (*maroon*), external globus pallidus (*blue*), and internal globus pallidus (*green*)) are displayed for reference using the DBS Tractography Atlas [68]. **(B)** Schematic representation of data acquisition: Metabolic imaging with [^18^F]-fluorodeoxyglucose (FDG) PET were conducted in PD patients with implanted STN electrodes on two consecutive days after 12 hours of medication washout on each day. On one day, participants were scanned off-stimulation (OFF) after ceasing stimulation for approximately three hours. On the other day, scanning was performed on-stimulation (ON), at the individual’s usual DBS settings. In a subset of patients, scans of regional cerebral blood flow (closely correlated with glucose metabolism) were additionally acquired using [^15^O]-water (H_2_^15^O) PET at multiple DBS settings in a single imaging session (see text). These scans were used to assess the effects of varying stimulation amplitude on network expression. **(c)** After image preprocessing, hemispheres with large stimulation-mediated improvement in contralateral limb motor ratings were selected for network identification using a supervised PCA approach applied to the FDG PET images (see Methods). Expression values for the resulting STN stimulation network (STN StimNet) were computed on a hemisphere-by-hemisphere basis in the ON and OFF conditions for individual patients in the network identification sample and in an independent validation sample.

**Figure 2:**
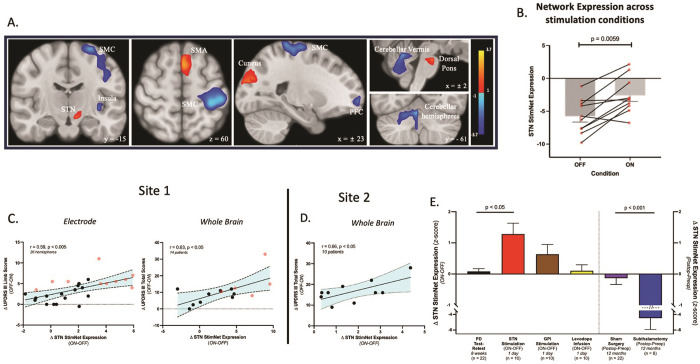
Coronal, axial, and sagittal view of a T1W MRI template showing areas with significant hemispheric contributions to the STN StimNet topography (Table 1). The network was characterized by stimulation-mediated increases (*red*) in the subthalamic complex (STN/substantia nigra), supplementary motor area (SMA), pons and cuneus. As part of the network, stimulation-mediated reductions (*blue*) were observed in the sensorimotor cortex (SMC) and cerebellar (vermian and paravermian structures). [Regions were displayed if the corresponding voxel weights contributed significantly to the network (│z│≥ 1.0) and were considered reliable on bootstrap estimation (inverse coefficient of variation (ICV) │z│≥ 1.65, p<0.05; 1000 iterations).] **(b)** STN StimNet expression values showed increases in nine of the 10 hemispheres used to identify the network (p=0.0059; permutation test) (see text). **(c)** *Left*: In the complete Site 1 dataset (see Methods), stimulation-mediated changes in hemispheric network expression ipsilateral to the electrode correlated with improvements in contralateral limb UPDRS motor ratings (r=0.59, p<0.005; Pearson correlation). *Right*: Analogous correlations were seen between whole brain network expression and whole-body motor ratings (r=0.63, p<0.05). [Values from scans used for network identification (see text) are displayed by red symbols; those used for in-sample testing are displayed by black symbols.] **(D)** For out-of-sample validation, we computed whole brain STN StimNet expression values ON and OFF stimulation in the independent Site 2 dataset (see text). As in Site 1, changes in these values correlated with improvement in total motor UPDRS ratings (r=0.66, p<0.05). **(E)** *Left*: Treatment-mediated changes in STN StimNet expression were greatest for STN-DBS (*red*), which differed significantly (p<0.05) from test-retest reliability of the measure (gray), whereas network modulation was comparatively modest for GPi-DBS (*brown*) (see text). By contrast, intravenous levodopa infusion (*yellow*) titrated to a level of motor benefit similar to STN-DBS (39.4% and 43.8% reductions in UPDRS motor ratings for the two interventions; see Table S1A) did not alter STN StimNet expression compared to test-retest (p>0.9). *Right*: Comparable motor benefit was also observed 12 months after unilateral subthalamotomy (*dark blue*) (see Table S1A). That said, STN StimNet expression declined rather than increased following the procedure, with the changes differing significantly (p<0.001; Mann-Whitney test) from those observed 12 months after sham surgery (*purple*).

The details of DBS electrode placement and the study design are illustrated in Fig. 1A and B (see Methods). We applied ordinal trend analysis to FDG PET scans from Site 1 acquired on and off STN stimulation in 10 hemispheres with large therapeutic responses in the contralateral limbs (see Methods). A significant ordinal trend was seen in the PCA data (Fig. 2B), with consistent stimulation-induced increases in pattern expression in 9 of 10 hemispheres (p < 0.01; permutation test). The topography of the resulting STN-DBS-related metabolic covariance pattern (STN StimNet) (Fig. 2A) was characterized by stimulation-induced metabolic increases (*red clusters*) in the subthalamic complex (STN/substantia nigra), supplementary motor area complex (SMA, BA6), cuneus (BA17), and dorsal pons. These changes were accompanied by stimulation-induced reductions (*blue clusters*) in the sensorimotor cortex (SMC, BA4/3), prefrontal cortex (BA9) and insula, and in the cerebellar vermis and hemispheres (lobules II, IV, V and the medial aspect of lobule VI) (Table 1). Voxel weights in the respective clusters were found to be reliable by bootstrap resampling (│*z*│≥ ±1.64, p < 0.05, one-tailed; inverse coefficient of variation (1000 iterations)).

We next prospectively computed STN StimNet expression on- and off-stimulation in the 14 remaining Site 1 hemispheres, i.e., those not used for network identification, and a significant ordinal trend was confirmed for the whole group (p < 0.001; permutation test). While concurrent changes in motor ratings were not used to derive the network, significant correlations were observed between stimulation-induced increases in hemispheric STN StimNet expression and improvement in contralateral limb performance (r = 0.59, p < 0.005; Pearson correlation, two-tailed) (Fig. 2C, *left*), and between corresponding changes in whole-brain network expression values and total motor ratings for the whole body (r = 0.63, p < 0.05; Pearson correlation, two-tailed) (Fig. 2C, *right*). The stability of these relationships was supported by the excellent test-retest reliability (ICC = 0.97) of the STN StimNet subject scores over an 8-week interval in PD patients (n = 22).

Lastly, we determined whether the observed changes in STN StimNet expression were reproducible in an independent group of PD patients who had undergone STN-DBS surgery (Site 2, see Methods). These individuals likewise underwent FDG PET on- and off-stimulation on consecutive days. In this patient sample, stimulation-induced increases in network expression were observed (p < 0.05; Student’s paired *t*-test, two-tailed), which correlated with concurrent improvements in total motor ratings (r = 0.66, p < 0.05; Pearson correlation, two-tailed) (Fig. 2D).

### Specificity of treatment-induced network changes

To determine the specificity of the STN StimNet for PD interventions of comparable clinical efficacy, we computed changes in STN StimNet expression in a number of treatment cohorts (Fig. 2E). For example, levodopa administration (*yellow bar*) acts by repleting nigrostriatal dopamine levels. Despite similar clinical responses, this treatment had no effect on STN StimNet expression (p > 0.9, compared to test-retest; Welch’s ANOVA, post-hoc Dunnett T3). Stimulation of the internal globus pallidus (GPi) (*brown bar*), located downstream from the STN, is also clinically effective. However, unlike STN stimulation (*red bar*), GPi-DBS does not significantly modulate network expression levels (adjusted p = 0.3, compared to test-retest; Welch’s ANOVA, post-hoc Dunnett T3). While stereotaxic STN lesioning (*blue bar*) can also be therapeutically effective, this procedure reduced rather than increased network expression levels compared to sham surgery (p < 0.001, Mann-Whitney test). In aggregate, the data points to this network as a specific therapeutic marker for STN-DBS in PD patients.

### Changes in network expression induced by varying stimulation intensity

**Figure 3:**
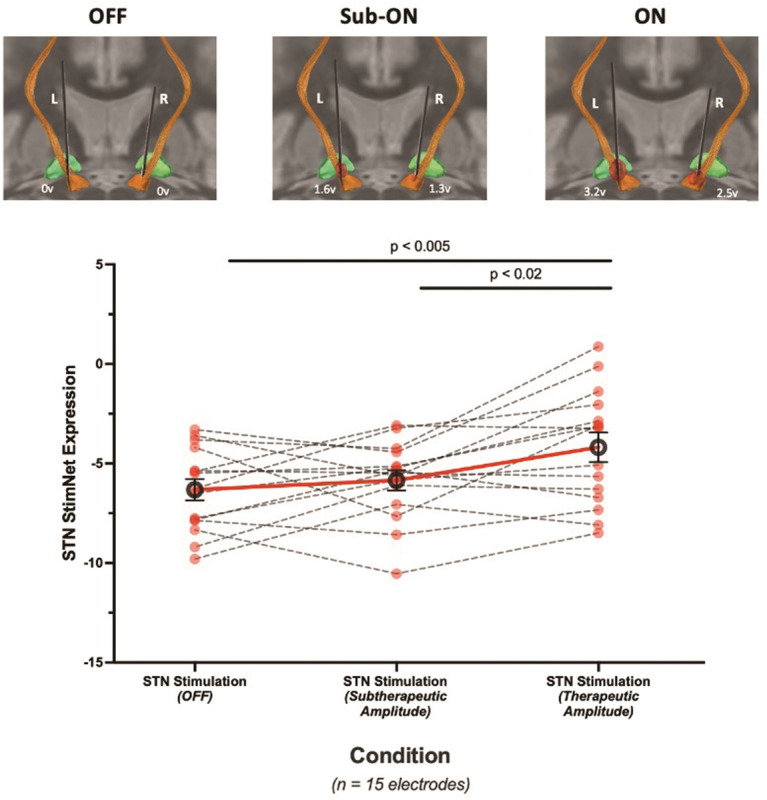
**(A)** Larger volumes of tissue activated (VTA, *red*) were observed with increasing stimulation voltage (keeping pulse width and frequency constant; see Methods) in a representative PD patient with bilateral STN-DBS. Subtherapeutic (Sub-ON) and therapeutic (ON) voltages in the left and right electrodes gave rise to progressively larger VTA within the STN (*orange*) and along adjacent white mater tracts (*orange fibers*). **(b)** Increasing stimulation voltage within the range of clinically optimized parameters for each patient (See Methods) resulted in consistent elevations in STN StimNet expression relative to the baseline (OFF) condition (p<0.001; one-way RMANOVA). [Circles denote the mean STN StimNet expression (± SE) for each condition. Differences in mean values across conditions are represented by solid red lines; individual patient data are represented by dashed black lines.]

Optimal selection of stimulation parameters is fundamental to maximize therapeutic benefit from STN-DBS. Too low a current delivered to the active contacts on the electrode is generally insufficient to modulate the STN (Fig. 3A), whereas excessively high voltages can lead to volumes of tissue activated (VTA) beyond the target, potentially leading to troubling side effects (Fig. S1C). To determine the relationship of stimulation intensity to changes in STN StimNet expression, we used [^15^O]-water (H_2_^15^O) PET to rapidly measure hemispheric STN StimNet expression in scans of cerebral blood flow (CBF) (duration 40 seconds) acquired in 12 PD patients (23 hemispheres) under varying stimulation conditions in the same imaging session (see Methods). In keeping with our previous findings, measurements of network expression computed in CBF scans correlated closely (r = 0.89, p < 0.001; Pearson correlation) with corresponding values computed in scans of cerebral glucose metabolism obtained with FDG PET in the same subjects (Fig. S1A) [17]. Indeed, in the CBF scans, higher stimulation voltages were associated with larger increases in STN StimNet expression relative to the baseline off-stimulation (OFF) condition (r = 0.42, p < 0.05; Fig. S1B). In a similar vein, we computed expression values for each hemisphere in the baseline (OFF), subtherapeutic (Sub-ON) and therapeutic (ON) conditions (15 hemispheres), and in the supratherapeutic (Supra-ON) conditions (8 hemispheres). Stimulation conditions for each subject were defined with reference to the individual’s usual stimulation parameters; voltage was varied while keeping frequency and pulse width constant (see Methods). Significant increases in STN StimNet expression were observed as stimulation intensity rose across conditions (F = 10.1, p < 0.001; RMANOVA) (Fig. 3B): values in ON were higher than OFF (p < 0.001, post-hoc adjusted Bonferroni correction) or Sub-ON (p < 0.05, post-hoc adjusted Bonferroni correction). That said, STN StimNet expression declined as stimulation intensity increased from the ON to the Supra-ON conditions (X^2^(2) = 7.0, p < 0.05; Friedman test with post-hoc Dunn’s test) (Fig. S1D).

### Baseline network expression predicts stimulation-mediated motor benefit

**Figure 4:**
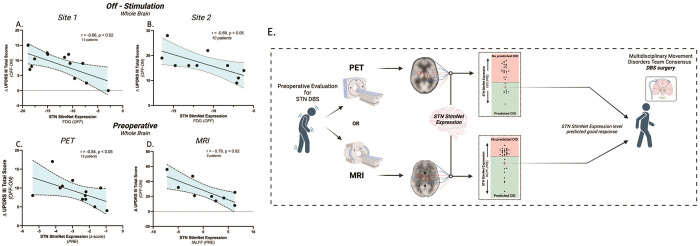
**(A)** STN StimNet expression measured off-stimulation (OFF) in each of the Site 1 patients correlated with stimulation-mediated motor improvement in total motor ratings for the same subjects (r=−0.66, p<0.02, Pearson correlation). **(B)** A similar significant relationship between OFF StimNet expression and improvement in total motor ratings was seen in the Site 2 validation sample (r=−0.68, p<0.02; see text) was also seen. **(C)** To assess correlations between stimulation-mediated motor improvement and preoperative (PRE) measurements of STN StimNet expression, we quantified this measure in 12 Site 1 PD patients who underwent FDG PET on average 8 months before bilateral STN-DBS surgery (see text). A similar correlation was observed for preoperative network expression and motor improvements seen with stimulation after DBS surgery (r=−0.54, p<0.05). **(D)** To assess the feasibility of using analogous measurements of network expression from BOLD imaging we quantified this measure in nine Site 1 PD patients who underwent rs-fMRI as part of their routine preoperative workup on average 17 days before bilateral STN-DBS surgery (see text). As with the PET cohort, preoperative STN StimNet expression correlated with stimulation-mediated motor improvements measured an average of 6 months after surgery (r=−0.79, p<0.02). **(E)** A schematic representation of the potential utility of preoperatively quantified network expression in clinical decision making. Patients that are being considered for STN-DBS surgery would undergo baseline scanning with either FDG PET or rs-fMRI imaging. STN StimNet expression levels computed using either imaging modality can then be considered in determining the patient’s suitability for DBS surgery.

With the reproducible relationship between the motor response to STN stimulation and STN StimNet modulation, we considered the possibility that preoperative measurements of network expression predict clinical outcomes in PD patients. This was motivated by the significant correlation that was observed between stimulation-mediated motor benefit and off-stimulation measurements of network expression in both STN-DBS groups (Site 1: hemisphere: r=−0.52, p < 0.01 (Fig. S2A); whole brain: r=−0.66, p < 0.02 (Fig. 4A); Site 2: whole brain: r=−0.68, p < 0.05 (Fig. 4B)). Accurate predictions of motor responses to stimulation were also observed in FDG PET scans acquired preoperatively (PRE), i.e., *before* electrode implantation surgery. Indeed, in 12 Site 1 patients, preoperative STN StimNet expression values obtained an average of 8 months before DBS surgery, correlated with motor benefit recorded approximately 17.3 months after surgery (hemisphere: r=−0.73, p < 0.0001 (Fig. S2B), whole brain: r=−0.54, p < 0.05 (Fig. 4C)).

Given the ease of acquiring rs-fMRI scans as part of the workup of PD patients, we explored the possibility that predictions of stimulation-mediated motor benefit can also be obtained by measuring STN StimNet expression preoperatively using resting-state blood oxygen level dependent (BOLD) signal imaging. To this end, we acquired rs-fMRI from nine patients on average 17 days prior to STN-DBS surgery. STN StimNet expression was computed in scans of fractional amplitude of low frequency fluctuations (fALFF) derived from the original time-series data. We found that the resulting values significantly correlated with the motor benefit recorded independently in these individuals approximately 6 months after surgery (whole brain r=−0.79, p < 0.02 (Fig. 4D), hemisphere: r=−0.49, p < 0.05 (Fig. S2C)).

We also examined the possibility of improving predictive accuracy by adding structural information regarding electrode placement and volume of tissue activated (VTA) with respect to the predefined STN “sweetspot” (Fig. S5B) [18]. Within the Site 1 rs-fMRI cohort, preoperative STN StimNet expression and postoperative lead location together predicted the magnitude of motor improvement with very high accuracy (F(2,6) = 7.219, p < 0.03, R^2^ = 0.7). Indeed, controlling for electrode placement, the contribution of STN StimNet expression to the model remained significant (β=−0.625, p < 0.05).

#### Network expression can be used to determine the optimal time for STN-DBS surgery in PD patients

**Figure 5:**
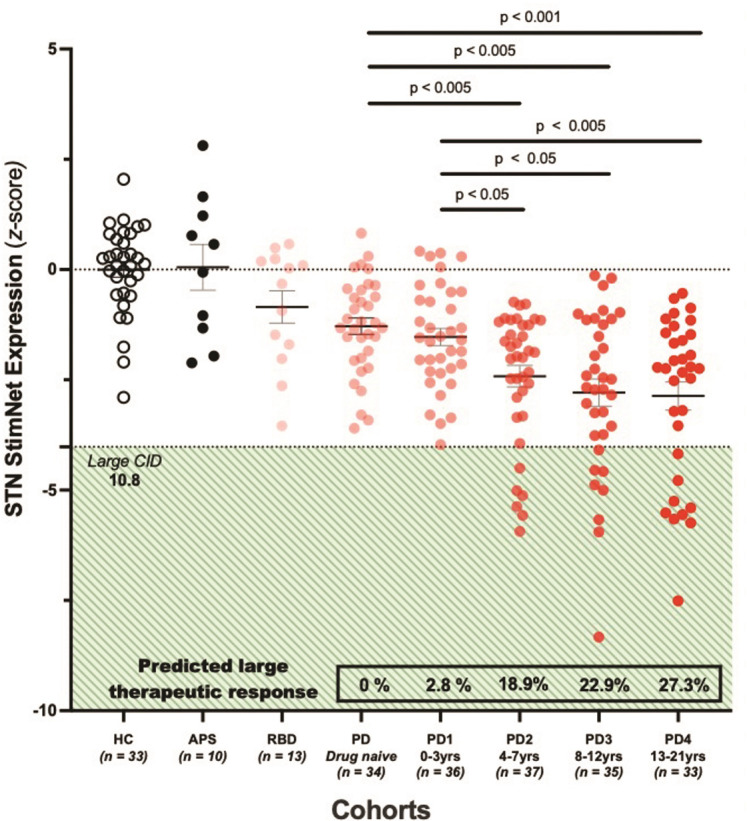
To demonstrate the potential use of baseline STN StimNet expression as a predictor of the clinical response to stimulation, we computed this measure in FDG PET scans from a population of 175 PD patients with disease duration ranging from 0–21 years from the time of diagnosis (see text). We found that network expression declined with increasing duration (p<0.001; ANCOVA, corrected for age). Low baseline expression levels, denoting increased likelihood of stimulation-mediated clinical benefit, became prominent over the first two duration blocks, without further decline in subsequent blocks (p>0.9; post-hoc comparisons, Bonferroni correction). Based on the regression model, the percentage of patients predicted to have a large clinically important difference (CID) response to STN stimulation (change in UPDRS motor ratings ≥10.8 points; see Methods) increased beginning at approximately 4 years after diagnosis, ranging from 18.9% in the second block (4–7 years) to 27.3% in the final block (13–21 years). Of note, low levels of baseline STN StimNet expression were not seen in patients with autopsy-confirmed atypical parkinsonian syndrome (APS), individuals with REM sleep behavior disorder (RBD), or untreated drug naïve early-stage PD patients. These conditions are thought to be either refractory or too early for DBS surgery.

With clinical trends in use of STN-DBS in earlier stages of PD, we explored the possibility that network expression can be used to identify optimal timing of surgery in individual patients [19]. To this end, we prospectively computed STN StimNet expression in 175 PD patients who were scanned with FDG PET between 0 and 21 years after the time of diagnosis (Fig. 5). The patients were grouped into blocks according to disease duration. Those with short and intermediate duration were divided into roughly three 4-year blocks (0–3 years, 4–7 years, and 8–12 years); those with long duration were grouped into a single 8-year block (13–21 years) (see Methods). Using individual patient STN StimNet expression and the regression equation that relates these values to stimulation outcome (Fig. 4C), we predicted the potential motor response to DBS for each subject and duration block. Additionally, the predicted motor outcomes were used to estimate the percentage of individuals in each block for whom the expected clinical response justified the procedure [20] (see Methods). The results were compared to a group of 33 age-matched healthy control (HC) subjects, 34 untreated early-stage PD patients who were naïve to dopaminergic medications, 10 autopsy-confirmed patients with atypical parkinsonian syndromes (APS) who are generally unresponsive to STN-DBS, as well as 13 individuals with REM sleep behavior disorder (RBD), a preclinical/prodromal syndrome associated with later conversion to PD or diffuse Lewy body disease (DLB) [21]. We found that baseline STN StimNet expression was reduced in the PD population compared to healthy control subjects (F(5,195) = 17.937, p < 0.001; one-way ANCOVA, adjusted for age). Analysis across duration blocks reveals declining network expression with advancing PD (F(3,136) = 4.983, p < 0.005; one-way ANCOVA, adjusted for age p < 0.001). Notably, reductions in network expression did not increase in magnitude beyond the second PD block (duration 4–7 years) (adjusted p > 0.9; post-hoc Bonferroni tests). By the same token, the proportion of patients expected to have substantial clinical improvement (i.e., large CID) in response to potential STN-DBS rose incrementally from 2.4% for 0–3 years to 27.7% for 13–21 years (p < 0.05, Fisher’s exact test). By contrast, network expression in RBD did not differ from normal (adjusted p > 0.9; post-hoc Bonferroni tests). Likewise, values in autopsy-confirmed APS patients were marginally elevated rather than reduced, denoting lower likelihood of clinical benefit compared to PD patients after the four-year mark (adjusted p < 0.001; post-hoc Bonferroni tests). Finally, drug naïve PD patients had similar network expression values compared to their treated age- and duration-matched early PD counterparts (duration ≤ 3 years) (p > 0.9; post-hoc Bonferroni tests). In this group, none of the patients had STN StimNet expression consistent with a large CID response to potential stimulation.

## Discussion

In this study, we identified and validated a network biomarker of the STN-DBS treatment response in PD patients. This network, termed STN StimNet, was treatment-specific in that it demonstrated greater modulation with STN-DBS compared to other antiparkinsonian interventions. We additionally found that stimulation-mediated changes in network expression correlated with concurrent motor benefit in the two independent groups of PD patients with implanted STN electrodes. Moreover, STN StimNet responses measured in a single patient under varying stimulation settings were greatest when parameters were optimized for maximal motor improvement without side effects. Notably, a consistent predictive relationship was observed between preoperative network expression levels for this network and stimulation-mediated motor outcomes recorded after DBS electrode implantation. Notably, preoperative measurements of STN StimNet expression obtained non-invasively from BOLD images confirmed the predictive relationship with DBS-mediated motor outcomes six months after surgery. Based on these relationships, we show how the preoperative STN StimNet measurements can be used *a priori* to identify those patients likely to receive the greatest and most meaningful clinical benefit from STN stimulation.

The STN StimNet network had only marginal spatial overlap with the established Parkinson’s disease-motor-related pattern, termed PDRP (Fig. S3) [14, 22]. While stimulation-mediated improvement in motor ratings is associated with reductions toward normal in PDRP expression, the latter changes are not intervention specific [15, 16, 23, 24]. Likewise, different relationships with disease duration were seen cross-sectionally for the two networks (Fig. S4). In contrast to the stepwise increases in PDRP expression seen with increasing disease duration, STN StimNet values began to approach a floor four years from the time of diagnosis [24–26].

The observed differences in expression values for the two networks can be explained by their distinct topographies. The STN StimNet includes a group of connected regions that are modulated by stimulation which are not necessarily affected (either directly or indirectly) by the underlying disease process. This included regions with negative weights (*blue clusters* in Fig. 2A) localized to the sensorimotor cortex (SMC) and to the contralateral cerebellar hemisphere and vermis, which denoted stimulation-induced reductions as part of the network. Elements of these regions also contribute to the PDRP (*yellow clusters* in Fig. S3), reflecting increased metabolic activity as part of the disease. These areas likely mediators of motor symptoms of PD, particularly tremor [27, 28]. By the same token, the STN StimNet also contains regional elements with positive weights (*red clusters* in Fig. 2A), which denote areas in which STN stimulation increased local metabolic activity as part of the network. These regions include the subthalamic complex (the STN and adjacent substantia nigra) and its projection ventrally to the pontine nuclei. Positive region weights on the network were also observed in the SMA complex (preSMA and SMA), perhaps as a reflection of antidromic stimulation of the hyperdirect pathway [29–32]. Interestingly, the relevance of this region to the STN StimNet topography has been demonstrated in recent studies in which improved connectivity along pathways linking these regions to the STN stimulation target in PD patients was associated with greater symptom relief, especially for akinesia and rigidity [32–34].

Apart from the relationship of stimulation-mediated changes in STN StimNet expression and clinical improvement, we found that the increase in network levels was greatest using the patient’s optimal ON parameters compared to measurements conducted with subtherapeutic (Sub-ON) or supratherapeutic (Supra-ON) settings. Selection of the appropriate stimulation settings is typically carried out on a trial- and-error basis. Monopolar review helps with the initial selection of appropriate electrode contacts; further fine-tunning is performed based on the alleviation of symptoms and the side effect profile [35, 36]. This may entail multiple office visits, given that acute motor responses during tuning sessions do not necessarily guarantee equivalent outcomes under more naturalistic conditions [36]. The current findings suggest that multiple within-session assessments of STN StimNet expression can potentially be used to identify optimal stimulation parameters in a more efficient manner. This approach requires rapid network measurements such as the H_2_^15^O PET technique employed in this study. Other imaging methods such as resting-state fMRI may also be suitable [15], given recent technical advances that allow for safe MR imaging protocols in DBS patients [37, 38]. In this regard, machine learning classifiers may also aid in the selection of optimal stimulation parameters using BOLD signal activation paradigms [39].

The exact location of the electrodes in relation to the STN target and adjacent motor pathways is critical for an optimal clinical response to DBS, as is the selection of ideal stimulation parameters [18]. Even so, other baseline clinical descriptors such as age, cognitive performance, and severity of motor disability, have been shown to correlate with postsurgical outcomes. In routine clinical practice, the levodopa challenge test (LCT; ≥ 33% improvement in motor symptoms with oral levodopa/carbidopa) is considered the gold standard for appropriate patient selection [40]. In this regard, the LCT is thought to provide an estimate of the maximum benefit a patient is likely to experience from STN stimulation. The cutoff criterion for the LCT has been found to vary considerably, and patient stratification according to this measure can be unreliable [41–43]. An unexpected finding in this study was the correlation seen at both sites between stimulation-mediated outcomes and STN StimNet expression recorded in the baseline OFF condition. Indeed, a similar correlation was observed in a separate Site 1 patient sample between DBS motor outcomes and STN StimNet expression levels measured in a baseline FDG PET scan obtained an average of 8 months before surgery. These relationships were all consistent: the PD patients with the lowest baseline values experienced the largest increases in network expression during stimulation, and accordingly the greatest clinical benefit. It is also noteworthy that analogous predictions of clinical outcome were not observed with PDRP expression values measured either preoperatively or in the OFF condition after DBS surgery. This relationship was maintained when STN StimNet measurements were calculated from functional MRI scans. Measurements of the relative contributions of fluctuations in the amplitude of BOLD signal seen within specific frequency bands with respect to the entire detectable range are termed fALFF and can give insights about the abnormal functioning of neurons in diseased states [44]. There exists a high bioenergetic coupling in regional glucose utilization (measured through FDG PET) and the fALFF signal [45, 46]. Therefore, perturbations in neural transmission are similarly reflected across both modalities [47]. In this regard, we found a similar relationship between preoperative STN StimNet expression and postoperative motor improvements in the rs-fMRI cohort. Network expression in these patients were measured from five-minute rs-fMRI sequences acquired during their routine preoperative clinical scans without the need for any additional visits. Therefore, replication of these results with an MRI based modality will likely allow for a more direct implementation of this STN stimulation specific marker into routine clinical practice.

While disease modification effects have been attributed to STN-DBS, the intervention is largely symptomatic: efficacy is gauged by improvements in standardized motor ratings. The patient, however, may or may not regard the rating changes as clinically meaningful. This is particularly relevant to surgical interventions such as STN-DBS, in which the inherent upfront risks of invasive electrode implantation and the cost of the procedure require relatively greater clinical improvement (i.e., a large CID) to be considered clinically justified [20]. Conversely, given the limited availability of DBS procedures and related services, even in affluent societies, prioritizing those patients likely to derive the greatest benefit becomes a reasonable option. The current study suggests that the proportion of patients with clinically meaningful outcomes may potentially be enhanced through preoperative metabolic imaging and the measurement of STN StimNet expression on an individual basis (Fig. 4E). That said, appropriate lead placement is fundamental to outcome, given that a misplaced or suboptimally placed electrode is unlikely to be effective irrespective of how well-suited the patient was for the procedure. Beyond its utility in patient selection, STN StimNet expression may have relevance to the management of treatment failure after DBS surgery. If a good outcome is predicted based on preoperative network expression, one may wish to re-adjust the DBS tuning parameters, or even reposition the leads – given that errors of as little as 2 mm can have substantial effects on clinical outcome [48]. A systematic study of preoperative network expression, lead placement/VTA, and other relevant predictors in a large patient sample will be needed to confirm these observations.

More generally, measurements of STN StimNet expression provide a unique perspective on optimal time for DBS surgery in PD. In the past, DBS has been reserved for patients with moderately advanced motor symptoms, for whom dopaminergic treatment had become ineffective or was accompanied by intolerable side effects, or both. That said, in recent years, the trend has shifted towards earlier surgery so as to reduce the frequency and severity of motor fluctuations, and other complications of chronic levodopa treatment [19]. To this end, we sought to determine the earliest time from diagnosis that STN-DBS is predicted to provide meaningful benefit to a substantial number of patients. We therefore computed STN StimNet values in 175 PD patients who were scanned with FDG PET between 0 and 21 years after clinical diagnosis (Fig. 5). While scanning was performed for research purposes unrelated to STN-DBS, we used the individual network data to estimate the proportion of patients in each duration block who were expected to have a large CID response to STN stimulation if surgery were performed in the 8 months that followed the imaging study (see Results). The findings were surprising in that expression levels began to plateau in the second 4-year block (4–7 years), with minimal further decline over the following 14 years. By contrast, PDRP values computed in the same patients continued to increase steadily over the same period (Fig. S4). We note that very few early-stage PD patients in the first block (0–3 years) would be expected to have a sufficiently large motor response to justify the surgery. With longer disease duration, the number of potential candidates increases slowly, reaching only 27% in the last block (13–21 years). In aggregate, with improvements in the safety profile of DBS surgery, the current findings accord with data from clinical trials suggesting that there is little advantage to delaying DBS surgery [19, 49–51].

Along similar lines, meaningful clinical responses to STN stimulation would not be expected in individuals with preclinical/prodromal disease (RBD) or newly manifest symptoms (drug naïve PD patients) who have yet to receive a course of oral dopaminergic therapy, or in patients with atypical parkinsonian syndromes (APS), in whom STN-DBS is known to be ineffective [52]. In contrast to patients with clinically established PD, the network data show that meaningful DBS responses are generally not expected at early disease stages, or, by the same token, when symptoms are caused by atypical variants of the disorder. Despite the relatively small numbers of autopsy-confirmed APS patients reported in this study, a marked difference in STN StimNet expression was present in this group compared to PD patients with clinical durations of 4 years or longer. That said, other network approaches exist for the accurate differential diagnosis of these conditions with FDG PET [14, 53, 54]

The current study has some limitations. The scans were performed for research as opposed to clinical purposes, which limited the size of the available datasets, particularly those acquired in multiple stimulation conditions. Along these lines, to identify a network relating to individual electrodes, the analysis was done on a hemisphere-by-hemisphere basis. While the behavior of each electrode was lateralized with regard to symptom relief, ON-state imaging was conducted at both sites with simultaneous deployment of the electrodes in both hemispheres. This strategy was pursued because of radiation safety considerations which prohibited acquiring ON scans in one hemisphere independently of the other. We recognize that our approach can potentially introduce confounding effects of stimulation-mediated spill-over from the opposite side. Nonetheless, we found that STN stimulation effects were sufficiently lateralized such that each side functioned independently with respect to hemispheric network modulation and changes in contralateral limb motor ratings. Indeed, relationships between hemispheric STN StimNet expression and contralateral limb motor outcome were maintained when corresponding measurements were computed for the whole brain. We also note that only a subset of participants had H_2_^15^O PET studies in the Sub-ON and Supra-ON conditions, so that inferences regarding associated changes in network expression levels should be made with caution. Likewise, structural imaging data on lead localization were only available in those patients with rs-fMRI imaging. This limited our ability to test predictive models that include the lead location in addition to the preoperative STN StimNet expression levels. Larger prospective studies will be needed to evaluate these measures as predictors of DBS outcomes in individual patients.

## Methods

### Patient demographics

We studied 43 patients with PD who underwent implantation of DBS electrodes into the subthalamic nucleus. Demographics and clinical characteristics for the two STN-DBS cohorts are summarized in Table S1A. Surgical technique in the Site 1 PET cohort (Northwell Health, Manhasset, NY, USA) and Site 2 cohort (San Raffaele Hospital, Milan, Italy) were similar and involved the use of a stereotaxic head frame and fused with high-resolution MRI for STN targeting and intraoperative confirmation using microelectrode recording and macrostimulation testing. Postoperative stimulation paradigms were adjusted by the patients’ neurologist to achieve maximal improvement in contralateral motor symptoms in the absence of side effects and stable prior to enrollment in the study.

### Imaging procedures

#### Site 1 (Manhasset, USA)

14 PD patients underwent PET imaging in the off- (OFF) and on- (ON) stimulation conditions on two consecutive days in random order with [^18^F]-fluorodeoxyglucose (FDG PET, cerebral glucose metabolism) and [^15^O]-water (H_2_^15^O PET, cerebral blood flow). On one of the days, stimulators were switched off approximately 3 hours before FDG PET and switched back on afterward. On the other day, FDG PET imaging was carried out in the on- stimulation condition. All patients fasted overnight (> 6 hours); antiparkinsonian medications were withheld for at least 12 hours before imaging. For each patient, motor ratings were evaluated according to the United Parkinson Disease Rating Scale (UPDRS III); ratings were separately recorded for each PET imaging session and stimulation condition. Schematic of imaging sessions and study design is presented in Fig. 1B.

FDG and H_2_^15^O PET were performed using the GE Advance tomograph (General Electric Medical Systems, Milwaukee, WI, USA) at The Feinstein Institutes for Medical Research (Manhasset, NY, USA) [55, 56]. The studies were performed in the resting state with eyes open in a dimly lit room. Ethical permission for the PET studies was obtained from the Institutional Review Board of Northwell Health. Written consent was obtained from each patient after detailed explanation of the procedures.

For validation of the network across imaging modalities a separate cohort of nine PD patients at Site 1 underwent rs-fMRI on the 3T Magnetom Prisma Siemens scanner as part of routine imaging before DBS surgery (Fig. S5). The resulting scans were compared to those from nine age- and sex- matched healthy volunteer subjects studied on the same platform (Fig. S5A). As with the PET cohort, UPDRS III ratings were recorded in the off-medication state (after at least 12 hours of washout) by a movement disorder specialist an average 4.8 months prior to STN-DBS surgery. Postoperatively, after a period of stimulation optimization, motor ratings recorded in the off-medication/on-stimulation condition were carried out on average 5.6 months after implantation.

#### Site 2 (Milan, Italy)

FDG PET was performed six months after STN-DBS surgery using the GE Discovery 1.5 PET/CT tomograph (General Electric Medical Systems, Milwaukee, WI, USA) at the San Raffaele Hospital (Milan, Italy). The imaging studies were conducted in the resting state after > 12 hours of medication washout. Scans in the stimulation ON and OFF conditions were acquired over two consecutive days as described elsewhere [57].

### Preprocessing

PET images from both sites were preprocessed with coregistration and spatial normalization to a standard FDG template [58]. To improve signal to noise, images were smoothed with an isotropic Gaussian kernel of 10 mm for FDG PET and 12 mm for H_2_^15^O PET [59]. Because STN stimulation is applied to each hemisphere independently, and predominantly affects the limbs contralateral to the electrode, network analysis was performed on each hemisphere (electrode) separately. We therefore created a hemispheric gray matter (GM) mask that included the midbrain, basal ganglia, thalamus, and cerebral cortex ipsilateral to the electrode, and the contralateral pons and cerebellum.

Preoperative MRI images from Site 1 were pre-processed using the FMRIB Software Library (FSL; http://www.fmrib.ox.ac.uk/fsl). Pre-processing included motion correction, brain extraction, spatial smoothing (kernel = 5 mm; FWHM) and high-pass filtering (cutoff = 100 s). The resulting fMRI volumes were registered to the individual subject’s structural T1 image and then to the standard Montreal Neurological Institute (MNI) 152 template. Scans were intensity normalized to reduce variability and improve the reliability. To compute the expression of the PET derived STN StimNet in the rs-fMRI time-series data we used the fractional amplitude of low-frequency fluctuation (fALFF) approach using the REST toolkit [44, 60].

### Network identification using Ordinal Trends Canonical Variates Analysis (OrT/CVA)

To identify a significant metabolic covariance topography associated with STN-DBS, we analyzed pairs of FDG PET scans acquired on- and off-stimulation on a hemisphere-by-hemisphere basis. This approach leveraged the side-to-side differences in response associated with STN-DBS and other surgical procedures for PD. Likewise, to maximize the signal induced by the intervention, the search for a significant STN stimulation network (STN StimNet) topography was limited to the 10 hemispheres (5 subjects) with significant improvement in contralateral limb ratings for the group as a whole.

To identify one or more metabolic covariance topographies associated with STN stimulation, we used Ordinal Trends Canonical Variates Analysis (OrT/CVA), a supervised principal component analysis (PCA) discussed in detail elsewhere (Fig. 1C) [14–16]. In contrast to disease-related metabolic networks, identified using combined data from patients and healthy subjects, OrT/CVA interrogates scans from individuals in different behavioral or therapeutic conditions, or in the same condition over time, for the presence of significant treatment-induced network topographies [16, 27, 61–64]. In brief, this approach identified sets of linearly independent (orthogonal) spatial covariance patterns for which individual expression values (principal component (PC) scalars) change consistently across conditions in all or nearly all subjects.

In OrT/CVA, expression levels for the candidate pattern are computed for each subject and condition in the derivation set. The search for a significant OrT/CVA topography was restricted to the top five PC patterns, together accounting for > 75% of the subject × voxel variance in the Helmert-transformed data matrix [61]. Subject scores for these PCs were then entered individually and in all possible linear combinations into a series of logistic regression models with treatment condition (stimulation ON/OFF) as the dependent variable. The simplest model that fits best to the data was selected according to the Akaike Information Criteria (AIC). The relevant PCs were linearly combined to yield a composite pattern, which was considered to be treatment-related if the associated subject scores exhibited a significant ordinal trend on permutation testing (p < 0.05, 1000 iterations). Voxel weights (PC loadings) on the pattern were evaluated by bootstrap resampling; the weight on a given voxel was considered reliable (i.e., not a reflection of outlier effects) for an inverse coefficient of variation (ICV│z│≥ 1.64, p < 0.05, one tailed; 1000 iterations) [64].

### Network validation

Expression values for the STN-DBS-related network (STN StimNet) were computed in prospective scan data using the topographical profile rating algorithm [65] (ScAnVP 7.0, available at www.feinsteinneuroscience.org). In this study, STN StimNet scores were computed for each hemisphere (implanted electrode) separately as well as for the whole brain. Only when comparing STN StimNet expression across cohorts (Figs. 2E, 4C *right* and 5), values were *z*-scored to a group of matched healthy controls (n = 33).

To assess the clinical significance of the network, stimulation-mediated changes in each patient were correlated with corresponding changes in UPDRS motor ratings for the contralateral limbs (sum of limb subscales for tremor, akinesia, and rigidity) and for the whole body (total motor score).

Independent validation was provided by the OFF/ON scan data from Site 2. Correlations between stimulation-related changes in network expression and whole-body motor outcomes were evaluated as above. For both cohorts, clinical-network correlations were evaluated as in Site 1 by computing Pearson correlation coefficients. The results in both groups were considered significant for p < 0.05.

For further validation, we examined the relationship of STN StimNet expression to stimulation intensity in the OFF, Sub-ON, ON, and Supra-ON conditions. This was done using [^15^O]-water (H_2_^15^O) PET to map cerebral blood flow (CBF) in a separate group of Site 1 PD patients with implanted STN-DBS electrodes (see Fig. 1B and Table S1A). Because the half-life of [^15^O] is only 40 seconds, the CBF scans in these patients were acquired in a single imaging session, with STN StimNet expression values computed for different stimulation parameters. The use of H_2_^15^O PET in this context is justified by the close coupling of network expression in H_2_^15^O and FDG PET scans acquired ON and OFF STN-DBS for PD-related metabolic networks [17] and the STN StimNet (Fig. S3A). By varying voltage and keeping pulse width and frequency constant, we scanned a subset of participants in either the subtherapeutic (Sub-ON, n = 15 electrodes) or supratherapeutic (Supra-ON, n = 8 electrodes) conditions in the same H_2_^15^O PET session as the ON and OFF conditions (Fig. 1B). Sub-ON was defined as the smallest reduction in stimulation voltage (relative to the patient’s clinically optimized settings) at which re-emergence of tremor was observed, whereas Supra-ON was defined as the smallest increase in voltage that was associated with stimulation side effects such as abnormal involuntary movements. Differences in network expression across OFF, Sub-ON, and ON stimulation conditions were evaluated with one-way repeated measures ANOVA (RMANOVA) with pairwise Bonferroni tests to correct for multiple comparisons. Differences in network expression across OFF, ON and Supra-ON stimulation conditions were evaluated with the Friedman’s test with pairwise Dunn’s tests to correct for multiple comparisons. Results were considered significant for p < 0.05.

### Treatment effects

To examine the specificity of STN StimNet as a treatment biomarker for PD, we evaluated the changes that occurred with other antiparkinsonian interventions: *(1) Levodopa infusion*: To compare the effects of STN stimulation with levodopa administration, we evaluated treatment-mediated changes in STN StimNet expression during each of the interventions. To this end, we compared 10 PD patients scanned on- and off-levodopa with a second group of 10 patients scanned on- and off-STN stimulation. The two groups were matched for age, gender, baseline UPDRS motor ratings, and for the change in motor ratings during treatment. *(2) GPi-DBS*: To evaluate the changes in network expression that were induced by stimulation at a different site along the basal ganglia-thalamocortical motor pathway, we measured STN StimNet expression in 10 patients who underwent FDG PET on- and off-GPi-DBS as reported previously [66]. *(3) STN lesioning*: To compare the changes achieved with STN stimulation with therapeutic ablation of the nucleus, STN StimNet values were computed in six previously reported PD patients who were scanned with FDG PET at baseline and 1 year after unilateral subthalamotomy [66, 67]. We also assessed the within-subject reproducibility of expression values for this network in PD patients who underwent test-retest evaluation with FDG PET over 8 weeks (n = 22) [55] or 52 weeks (n = 21) [16]. STN StimNet responses to STN- and GPi-DBS and with levodopa infusion were compared with test-retest reliability of the measure over 8 weeks [55]. The changes in network expression following unilateral subthalamotomy were compared to corresponding differences over 12 months in PD patients who underwent sham surgery (burr holes) as part of blinded gene therapy trial [16]. The demographic details and clinical characteristics of these groups are provided in Table S1A. Differences in treatment-mediated STN StimNet modulation across the test-retest levodopa infusion, STN- and GPi- DBS groups were evaluated using a Welch’s one-way ANOVA with post-hoc Dunnett’s T3 tests in relation to test-retest; the results were considered significant for p < 0.05. Differences in treatment-mediated STN StimNet modulation between the sham surgery and subthalamotomy groups were evaluated using the Mann-Whitney test; the results were considered significant for p < 0.05, two-tailed.

### Predictive models

To evaluate STN StimNet expression as a potential predictor of the clinical response to STN-DBS in PD patients, we examined the correlation between treatment-mediated improvement in motor ratings and baseline network measurements obtained OFF stimulation in patients with implanted electrodes. Relationships between the clinical response and OFF-state network expression were separately assessed in STN-DBS patients from Site 1 (n = 13) and Site 2 (n = 10). A separate group of Site 1 patients (n = 12) were scanned with FDG PET, an average of 8 months before STN-DBS surgery. To assess feasibility of measuring STN StimNet from MRI based BOLD imaging, we computed expression scores from fALFF images acquired an average 17 days before surgery (Table S1A). Preoperative expression levels from these group likewise correlated with stimulation-mediated motor changes recorded after electrode implantation. Correlations were considered significant for p < 0.05, Pearson correlations. To visualize the effects of varying stimulation amplitude on the individual VTA in relation to surrounding anatomical structures, we used the standardized pipeline in Lead-DBS V3.0 and the DBS Tractography Atlas [68, 69].

Lastly, we demonstrated how STN StimNet expression can be used preoperatively to identify those PD patients who are likely to benefit from STN-DBS surgery. We stratified potential STN-DBS-related outcomes according to the clinically important difference (CID), a method that links quality of life and other patient-reported outcomes to specific thresholds on the clinical rating scale. That is, by relating treatment-mediated changes in ratings to the patients’ perceived outcome, CID provides an index of clinical significance that is not available otherwise [20]. With invasive surgical procedures such as STN-DBS, which carry patient risk and societal expense, CID should be larger than for alternative pharmacological or other non-invasive interventions [70]. Prior analysis of blinded PD studies has shown that large CID equates to an absolute change of 10.8 in total UPDRS motor ratings [20]. To estimate the STN StimNet expression score that corresponds to a large predicted CID response with STN stimulation, we used the regression model presented in the text (Fig. 4C). To this end, we analyzed FDG PET scans from 175 Site 1 PD patients, who were divided into roughly 4-year blocks based on the time from clinical diagnosis [25, 26, 71]. For each patient, we computed whole-brain STN StimNet expression and standardized the measurements with respect to corresponding values from 33 healthy control subjects [72]. We compared the resulting values to those from individuals who are not considered to be DBS candidates for a variety of reasons: *(1) Preclinical syndrome*: STN StimNet expression was computed in FDG PET scans from 13 individuals (13M; age 63.5 ± 8.4 years) with REM sleep behavior disorder (RBD), a preclinical syndrome in which motor symptoms are not initially present but which frequently evolves into PD or a related movement disorder [73, 74]. *(2) Drug naïve PD*: Similarly, we computed network expression values in a group of early-stage PD patients matched in age and duration to those in the first PD block (0–3 years; Table S1B) but who were drug naïve, i.e., without prior exposure to dopaminergic medication [71]. Drug naïve PD patients are typically not referred to DBS surgery without an intervening period of chronic oral dopaminergic medication. *(3) Atypical parkinsonian syndromes*: Network expression was computed in scans from 10 patients (4M; age 66.3 ± 6 years) with autopsy-confirmed atypical parkinsonian syndromes (APS) such as multiple system atrophy (MSA) or progressive supranuclear palsy (PSP) [14, 75]. Patients with APS can be clinically confused with PD but are typically not suitable for STN stimulation [76]. Network expression values were compared across groups using one-way ANCOVA, with appropriate post-hoc contrasts.

### Statistical analysis

All statistical analysis and graphical plots were carried out using IBM SPSS Statistics for Windows, Version 28.0 (Armonk, NY, USA) and Graphpad Prism, Version 9.5.1 for Windows (Boston, MA, USA). Illustrations were prepared in BioRender.com.

## Figures and Tables

**Table 1:** 

Region	MNI Coordinates *Peak Voxel*			Region Weights *Peak Voxel* (z-score)
	x*	y	z	
STN stimulation mediated metabolic increases (ON > OFF)				
Subthalamic complex (STN)^a^	± 12	−16	−14	2.63
Dorsal pons	± 2	−20	−24	3.13
Supplementary motor area complex (SMA) (BA6)^b^	± 2	16	54	3.71
Cuneus (BA 19)	± 20	−98	20	3.11
STN stimulation mediated metabolic decreases (ON < OFF)				
Cerebellar vermis and hemispheres (lobules II, IV, V and the medial aspect of lobule VI)	± 2	−62	−20	−3.55
Sensorimotor cortex (SMC) (BA4/3)	± 34	−32	58	−3.6
Insula (BA13)	± 42	−10	−4	−2.7
Prefrontal cortex (PFC) (BA9)	± 24	64	−2	−2.83

* STN StimNet was a hemispherically derived network therefore regions are symmetrically represented on the left or right

a Subthalamic complex consists of the subthalamic nucleus and ventrally located substantia nigra

b Supplementary motor area complex consists of the SMA and pre-SMA
